# “No… Nobody told me anything about that”: a qualitative study on pretreatment knowledge of individuals with lung or head and neck cancer regarding the disease, treatments, and physical exercise

**DOI:** 10.1007/s00520-026-11407-1

**Published:** 2026-10-27

**Authors:** Isabela Duarte, Karina Arielle da Silva Souza, Alice Haniuda Moliterno, Paloma Borges, Matheus Pedroso, Isis Grigoletto, Ercy Mara Cipulo Ramos

**Affiliations:** 1 Department of Physiotherapy, School of Technology & Science (FCT/UNESP), São Paulo State University, Roberto Simonsen Street, 305, Centro Educacional, Presidente Prudente, São Paulo, 19060-900 Brazil; 2 Curtin School of Allied Health, Curtin University, Perth, WA Australia; 3 Curtin School of Nursing, Curtin University, Perth, WA Australia

**Keywords:** Lung neoplasms, Head and neck neoplasms, Knowledge, Health education, Exercise, Qualitative research

## Abstract

**Purpose:**

Lung and head and neck cancers have high incidence, substantial symptom burden, and marked functional decline related to both the disease and its treatments. Although physical exercise is recognized as an adjuvant strategy that mitigates treatment-related adverse effects, patients’ understanding of their diagnosis, therapeutic plan, and the role of exercise may affect their engagement and adherence. Therefore, this study aimed to investigate the knowledge of individuals with primary lung or head and neck cancer, prior to treatment initiation, regarding the disease, prescribed therapies, anticipated symptoms, and the role of physical exercise as an adjunct intervention.

**Methods:**

This qualitative study included individuals with primary lung or head and neck cancer. Data were collected through semi-structured individual interviews conducted up to 1 week before treatment initiation, recorded, and transcribed verbatim. Data collection continued until thematic saturation, and analysis followed Braun and Clarke’s six-phase thematic analysis approach.

**Results:**

A total of 14 individuals were interviewed (age, 66 [53–70] years; head and neck cancer, 8 (57%); males, 10 (71%)). Five overarching themes and 13 subthemes were identified from the data: (1) general knowledge; (2) expectations during the cancer treatment; (3) perceptions about physical exercise in cancer; (4) third-party support; and (5) context of the diagnosis.

**Conclusion:**

Individuals with lung or head and neck cancer, prior to treatment initiation, show limited knowledge about their disease, prescribed therapies, expected symptoms, and the role of physical exercise. Although participants had minimal understanding of what exercise programs entail, they expressed willingness to participate if recommended.

**Supplementary Information:**

The online version contains supplementary material available at https://doi.org/10.1007/s00520-026-11407-1.

## Introduction

Lung and head and neck cancers are among the most prevalent and lethal malignancies worldwide, responsible for high incidence and mortality rates [1, 2]. Both conditions are commonly associated with smoking and other risk factors such as alcohol consumption, occupational exposure, and air pollution [3–6]. The disease and its treatments frequently lead to physical, psychological, and social impairments, including fatigue, muscle weakness, reduced functional capacity, and diminished quality of life [7, 8].

Physical exercise has been widely recognized as a non-pharmacological strategy capable of mitigating several treatment-related symptoms, improving functional capacity, and enhancing quality of life in individuals living with and beyond cancer [7, 9–12]. Furthermore, the incorporation of physical exercise before the initiation of cancer treatment, referred to as prehabilitation, aims to improve adherence to and tolerance of oncological therapies. This strategy enables a comprehensive baseline physical and functional assessment, helps preserve functional capacity and quality of life, and may enhance long-term survival by reducing treatment-related side effects and toxicity. Prehabilitation is recommended to prepare patients for surgical procedures as well as for chemotherapy and/or radiotherapy. However, despite the well-documented benefits of physical exercise, adherence to exercise programs remains low due to disease-related symptoms, sociodemographic and cultural barriers, limited access to prehabilitation and rehabilitation services, and insufficient professional guidance [13–15].

Qualitative research has provided valuable insights into the experiences, attitudes, and barriers perceived by cancer patients regarding exercise during or after treatment. Themes such as symptom burden, the need for professional support, and the emotional benefits of exercise have been frequently reported [16–18]. However, most of these studies were conducted after treatment initiation, leaving a critical gap in understanding how individuals perceive their disease, planned therapies, expected symptoms, and the role of physical exercise before beginning oncologic treatment. Understanding these perceptions is essential for guiding patient-centered care and improving adherence to exercise-based interventions [19, 20].

Therefore, the primary aim of this study was to investigate the knowledge of individuals with primary lung or head and neck cancer, prior to initiating oncologic treatment, regarding their disease, prescribed therapies, expected symptoms, and the role of physical exercise as a complementary intervention. Secondary aims were to (i) explore patients’ perceptions of performing or not performing physical exercise associated with oncologic treatment and (ii) analyze their understanding of how exercise may influence treatment-related adverse symptoms.

## Methods

### Study population and design

This descriptive qualitative study was conducted in accordance with the guidelines of the Consolidated Criteria for Reporting Qualitative Research (COREQ) [21]. Participants were individuals with lung or head and neck cancer under clinical follow-up at the Hospital de Esperança de Presidente Prudente (SP), an oncology reference center serving 45 municipalities in the region. Eligible participants were adults (≥ 18 years) with histopathologically confirmed primary lung or head and neck cancer, scheduled to begin radiotherapy and/or chemotherapy, and with preserved cognitive and verbal ability. Exclusion criteria included cognitive impairment preventing communication, absence from three scheduled interview attempts, lack of access to a smartphone or internet for remote participation, and initiation of oncologic treatment before the interview. All participants provided written informed consent prior to inclusion.

Data were collected through individual interviews guided by a semi-structured script containing ten questions addressing knowledge about the disease, prescribed treatments, potential symptoms, and the performance of physical exercise during cancer treatment (Online Resource 1). Interviews were conducted up to 1 week before treatment initiation, either in person or via Google Meet®, according to participant availability. All interviews were conducted by the first author (female), who had no prior contact with the participants. The interviewer underwent specific training and conducted a pilot study with three interviews to become familiarized with the process, refine the question script, and establish the average interview duration, which was approximately 40 min. All interviews were audio- and video-recorded and transcribed verbatim after participants provided consent. The interviews were recorded to ensure that no verbal or non-verbal reactions expressed by the participants were overlooked during data collection and analysis. Data collection continued until thematic saturation [22] was reached, which occurred after 11 participants. Three additional interviews were conducted to confirm the saturation point, with no new codes or themes identified.

### Qualitative and quantitative analysis

Thematic analysis was performed according to the six interactive phases proposed by Braun and Clarke [23]. Theme development and refinement were conducted independently by three researchers (researcher triangulation), and discrepancies were resolved through consensus by comparing the assigned codes, discussing individual interpretations, revisiting the original transcripts, and reaching agreement either by creating a new code or selecting the most representative existing code. A hybrid coding approach was employed, beginning with codes derived from the literature while allowing new codes to emerge during the analysis of the interview transcripts. Ten percent of the transcripts were reviewed by the participants to confirm the consistency and validity of the identified themes. The participants’ names have been abbreviated in order to maintain their confidentiality. For the quantitative analysis of the sample characterization, descriptive statistics were used, and the data were expressed as median and interquartile range, and the categorical data were expressed as frequencies and percentages, analyzed using the Statistical Package for Social Sciences (SPSS) version 29.0 (IBM Corp, Armonk, NY, USA).

## Results

Fourteen individuals completed participation in the study. Among the 14 participants analyzed, most were male (71%), diagnosed with head and neck cancer (57%), and prescribed combined chemotherapy and radiotherapy treatment (65%). Additionally, only four participants (29%) reported previous experience with physical exercise. Table 1 presents the sample characterization data.

**Table 1:** Sociodemographic and clinical characterization of the study participants

Variables	Total sample (*n* = 14)
Sociodemographic data	
Age (years), median [IQR]	66 [53–70]
Sex (male), *n* (%)	10 (71%)
*Marital status, n (%)*	
Married	5 (36%)
Widowed	6 (43%)
Divorced	3 (21%)
*Educational level, n (%)*	
Incomplete elementary education	4 (29%)
Complete elementary education	3 (21%)
Complete high school	6 (43%)
Complete higher education	1 (7%)
ECOG performance status, *n* (%)	
0	6 (42%)
1	4 (29%)
2	4 (29%)
Oncological aspects	
***Tumor site, n (%)***	
Head and neck cancer	8 (57%)
Lung cancer	6 (43%)
***Prescribed treatment, n (%)***	
Chemotherapy	2 (14%)
Radiotherapy	3 (21%)
Chemotherapy + radiotherapy	9 (65%)
Lifestyle habits	
***Smoking status, n (%)***	
Smoker	3 (21%)
Former smoker	5 (36%)
Non-smoker	6 (43%)
***Previous experience with physical exercise, n (%)***	
Yes	4 (29%)
No	10 (71%)

Values are expressed as median, interquartile range (*IQR*), frequency, and percentage

*n* number, *m* male, % percentage, *ECOG* Eastern Cooperative Oncology Group performance status score

**Fig. 1:**
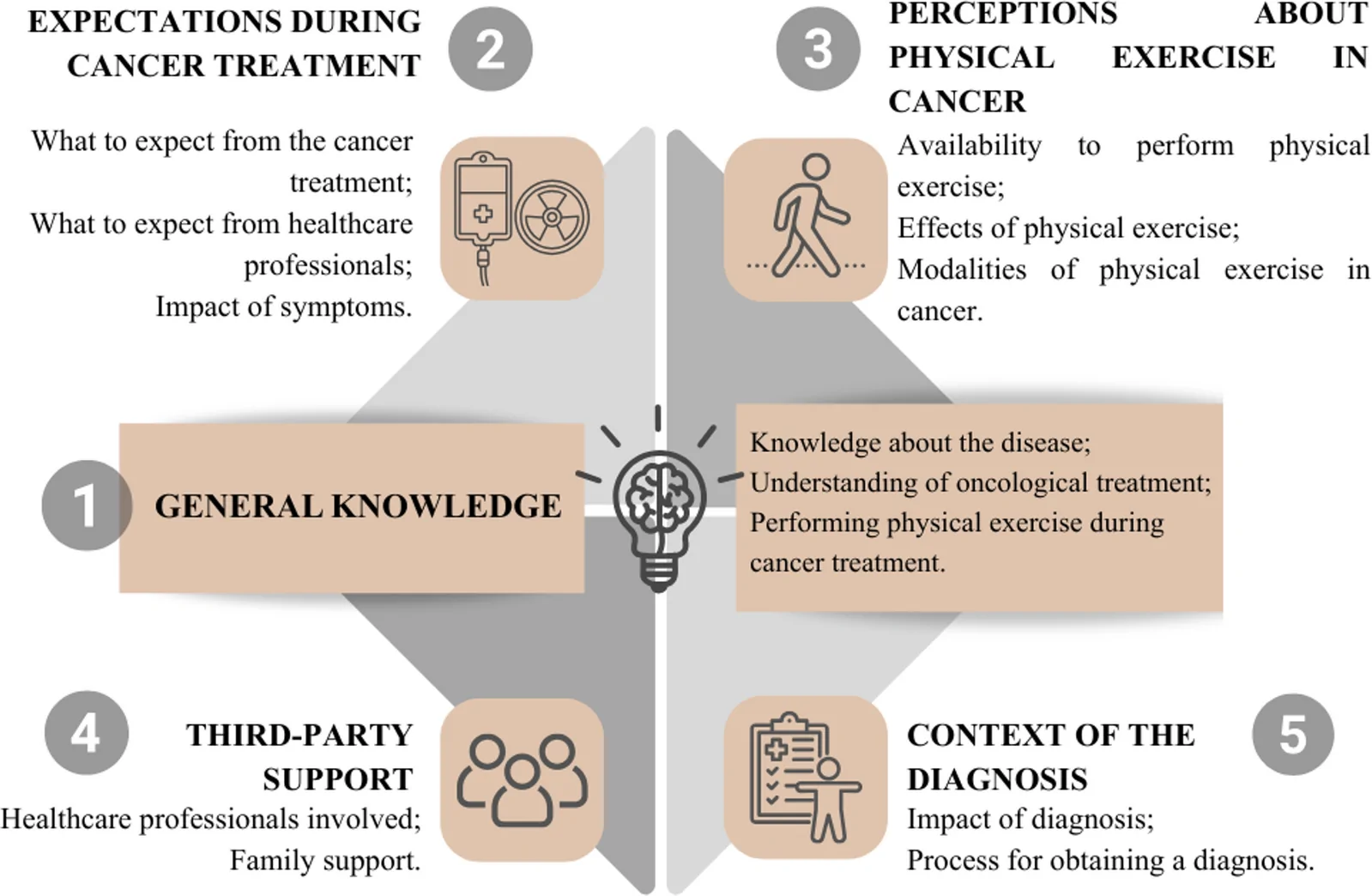
Themes and subthemes emerging from the thematic analysis

Thematic analysis revealed five main themes and 13 subthemes that emerged from the collected data. The corresponding themes and subthemes are presented in Fig. 1. The complete thematic analysis is provided in Online Resource 2.

### Theme 1: general knowledge

In this theme, three subthemes emerged that represent the participants’ overall knowledge: (i) *knowledge about the disease*; (ii) *understanding of oncological treatment* *and symptoms*; and (iii) *performing physical exercise during cancer treatment*. This theme encompasses what participants know about the disease, its treatment, associated symptoms, and the role of physical exercise in the oncological context.

Regarding *knowledge about the disease*, one participant demonstrated detailed understanding of cancer, while the others showed limited knowledge about the disease, its development, and the stage of cancer they were in. Most of the participants’ knowledge stemmed from experiences with relatives or close acquaintances who had been diagnosed with cancer.

Concerning *understanding cancer treatment and symptoms*, participants were generally aware of which therapy had been prescribed to them. However, a lack of understanding about how the treatment procedures are conducted predominated. Their comprehension of possible symptoms varied, but most listed several symptoms associated with the prescribed cancer treatment, expressing the expectation that these would not cause significant physical debilitation.

As for *performing physical exercise during cancer treatment*, two out of the 14 participants reported any knowledge of the possibility of performing exercise during cancer treatment. The remaining participants demonstrated a complete lack of awareness regarding this subtheme, and one mentioned that learning about it prior to beginning treatment would have been important.

Table 2 presents representative excerpts for each subtheme that emerged from the main theme 1.

**Table 2:** General knowledge and its subthemes

Theme	Subthemes	Representative excerpt
General knowledge	Knowledge about the disease	**R.C.N., female:** “I know they’re dead cells, right? They die, they clump together, they settle down, and that’s what happens, that’s what I know… they’re tumors that sometimes a person can’t even talk about.”
		**R.C.S.F., male: “**Oh honey, I can’t even say what I know, I only found out after June 10th.”
		**M.J.R., female:** “I’ve already lost an aunt, I’ve already lost a cousin… all to these diseases. So, what I can say is that it [the disease] is tragic, isn't it?”
		**J.A.S., male:** “Ah, I think it [cancer] is a disease, like mine, it’s a very aggressive disease and it ends up destroying a person’s health, it consumes their life, that’s what I think.”
		**A.S., male: “**Cancer, all [the symptoms].”
	Understanding cancer treatment and symptoms	**L.F.C., male:** “I’ve never been like that, I’ve never pulled so much stuff from the internet… because sometimes it might not be useful for me, so I prefer to live more in the moment, actually.”
		**R.C.N., female: “**I have no idea, because I’ve never seen it done, nor do I know what it’s like. I know there’s injectable chemo, there’s chemo that’s… in pill form, liquid, but I’ve never seen anyone take chemo, I’ve never seen anyone undergo chemotherapy treatment, so I don’t know, I don’t know what it’s like…”
		**J.P.V., male:** “It’s going to affect our bodies, right? I know it’s going to shake things up. It's not like it’s going to happen and then everything will be normal. When the treatment starts, it’s going to affect our bodies […] It’s weakness, right? Weakness!”
	Performing physical exercise during cancer treatment	**L.F.C., male:** “I haven’t heard of it because my research is very limited. And besides, to what extent will this patient be debilitated?”
		**R.C.N., female:** “I’ve heard that at the hospital they made me exercise while I was there.”
		**R.C.S.F., male:** “Ah, I can’t even speak, because I’ve never heard of it, right? If I ever had that perspective, I could even give an answer, but I’ve never heard of it.”
		**J.A.S., male:** “No… Nobody told me anything about that.”

### Theme 2: expectations during cancer treatment

In this theme, three subthemes emerged that reflect the expectations of individuals undergoing oncological treatment: (i) *what to expect from the cancer treatment*; (ii) *what to expect from healthcare professionals*; and (iii) *impact of symptoms*.

Regarding *what to expect from the cancer treatment*, participants emphasized that they believe the treatment will be beneficial, even though they recognize it will be challenging. One participant reported believing that the oncological treatment would not be effective. Faith was frequently identified in participants’ narratives, serving as a source of hope and confidence that the treatment would yield positive outcomes.

In relation to *what to expect from healthcare professionals*, all participants expressed trust in the medical and multidisciplinary team, with most believing that the professionals would perform their duties to the best of their abilities, even though some were uncertain about the specific roles or interventions expected from these professionals.

The *impact of symptoms* was a concern expressed even before the beginning of the cancer treatment. Most participants were already experiencing symptoms related to cancer and believed that these could worsen as a result of therapy. Their perceptions regarding potential symptoms were diverse, with most focusing on nutritional issues and physical weakness. They also reported that these symptoms would likely interfere with their daily routines, limiting their ability to perform usual activities as they did prior to treatment.

Table 3 presents representative excerpts for each subtheme that emerged from the main theme 2.

**Table 3:** Expectations during cancer treatment and its subthemes

Theme	Subtheme	Representative excerpt
Expectations during cancer treatment	What to expect from cancer treatment	**M.V., male:** “Because I’m going to have a lot of willpower, right? You have to have strength, hope, right? And not give up!”
		**A.S., male:** “Yeah… we’re going to feel bad […] we won’t be able to walk… Yeah, we won't be able to do anything with our lives. All our thoughts are negative.”
		**N.D., male:** “That’s why, sometimes, with all the resources we have today, we don’t worry too much, do we?”
		**J.P.V., male:** “If it doesn’t always cure it [cancer], sometimes it provides some relief… So, it’s a little relieving for us, right…”
	What to expect from healthcare professionals	**M.V., male:** “So you are capable. I believe in you too. I believe in God and in you! May He guide you so that everything goes well!”
		**L.F.C., male:** “Being consistent with the patient, don’t leave out any information, say things like ‘we’re doing this because of this and that,’ ‘the probability of a cure is this.’”
		**S.S.F., female:** “Ah, I expect everything. But I don’t know what ‘no’ means.”
		**R.C.N., female:** “I have complete faith in the doctor and in God (strong affirmation), I am certain that with their help and yours, I will get through this.”
	Impact of symptoms	**A.S., male:** “You can’t do it, you have to stay seated.”
		**C.O.P., male:** “At work, you know, you don’t know if you’ll be able to go back to work, what’s going to happen.”
		**N.G.S., female:** “I feel unwell, and my legs are kind of weak, you know? All day long… I feel fine sleeping, lying down like this I feel good, but even just walking around the house I’m trembling.”
		**N.D., male:** “Look, it can sometimes interfere with the emotional system. […] And look, if I continue the way I am with this muscular support, I believe it will get worse if I don’t take action, you understand?”

### Theme 3: perceptions about physical exercise in cancer

In this theme, three subthemes emerged that constitute the participants’ perceptions of physical exercise in the context of cancer: (i) *availability to perform physical exercise*; (ii) *the effects of physical exercise*; and (iii) *modalities of physical exercise in cancer*.

The subtheme “availability to perform physical exercise” encompasses participants’ perceptions regarding whether it is possible to engage in physical exercise after being diagnosed with cancer and whether they would be willing to do so during oncological treatment if proposed. In this subtheme, eight participants expressed uncertainty about the feasibility of exercising while having cancer: this uncertainty being associated with their expected physical debilitation during treatment. Six participants, however, believed that performing exercise would be possible, while the remaining individuals considered it unfeasible. When asked whether they would participate in an exercise program if recommended, two participants stated they would not, believing they would be physically unable to do so, whereas the others affirmed they would engage in the activities with the intention of “healing,” “improving,” or “following the professional team’s guidance.”

Regarding the subtheme “effects of physical exercise,” 11 participants described perceived positive outcomes on physical function, expressed through terms such as “to strengthen,” “to avoid muscle atrophy,” “to reinforce,” “to improve the body,” or “to be more active.” All 11 also mentioned emotional benefits, reporting that exercise would help with “the mind,” providing greater motivation and vitality. However, three participants expressed concern that exercise might worsen symptoms already experienced—particularly gastrointestinal discomfort, fatigue, pain, and cough—while one participant believed that exercise could interfere negatively with oncological treatment.

Concerning the subtheme “modalities of physical exercise in cancer,” 12 participants stated that the activities would probably consist of light exercises, with the type and intensity depending on their physical condition. Two participants mentioned specific exercise modalities, such as “dance,” “treadmill walking,” “balance exercises,” or “stretching.” Despite these mentions, uncertainty regarding the nature and intensity of the exercises was evident, as most participants were unsure of what these activities would actually involve. Additionally, two participants reported having no idea of what cancer-related exercises might consist of.

Table 4 presents representative excerpts for each subtheme that emerged from the main theme 3.

**Table 4:** Perceptions of physical exercise in cancer and its subthemes

Theme	Subtheme	Representative excerpt
Perceptions about physical exercise in cancer	Availability to perform physical exercise	**N.G.S., female:** “Can a person handle doing it? I don’t think so. […] A person gets weak, doesn’t they? I already feel terribly weak without doing any exercise, nothing.”
		**R.C.N., female:** “I don’t think I can (shakes his head no while answering), it will depend on what the doctor says, if she says I can, I’ll do it.”
		**C.O.P, male:** “Right now I don’t know if I would do it. […] Oh, I’m always afraid to do it, because it takes away a person’s stability a little, you know [the cancer].”
		**R.C.S.F., male:** Me? If they tell me to do it, then I’ll do it, because I want to get better.”
	Effects of physical exercise	**M.V., male:** “I believe it will give us more encouragement, right? We won’t give up.”
		**R.C.N., female:** “I think it can worsen [the symptoms] if there isn’t good follow-up, it can get worse, you know. Now, if it’s well monitored and the doctor says I can, I'll do it! […] I think that, if it’s light exercise, it won’t interfere, but if it’s heavier, it will interfere with fatigue, stomach problems, movement… That’s the idea I have. […] I think that instead of helping, it will hinder, right?”
		**N.D., male:** “I believe it would be like reinforcing the foundation of a house, you know? You have to reinforce the base, I think that’s exactly what it entails.”
		**J.A.S., male:** “I think that exercise, along with the treatment, the treatment will help me to get rid of the cancer and the exercise would, how do you say… help me to be more active, to stay in shape, to fight it [the cancer].”
	Modalities of physical exercise in cancer	**L.F.C., male:** “I don’t even have the slightest idea […] But now, what kind of neck exercise would that be (laughs)? Stretching?”
		**R.C.N., female:** “Ah, I don’t think it should be anything too heavy, it should be something light, because whoever is undergoing this kind of treatment isn’t exactly healthy, right? (laughs) I think it has to be something lighter… because the old lady can’t handle anything too heavy! (another laugh)”
		**N.D., male:** “I believe that it [exercise] is limited, you know. I believe that you can't be doing everything you want, right? You can’t, like, do a triathlon, you know? (laughs) […] I think the treadmill isn’t good, I think it’s too much effort. It would be more stationary and also in terms of balance.”
		**J.A.C., male:** “It’s not one of those exercises that involve lifting weights, you know? I don’t think it can be like that for cancer. It must be another type of exercise, right? A dance, something lighter. But then you’ll just get used to it, until your body gets used to it.”

### Theme 4: third-party support

In this theme, two subthemes emerged that represent the support offered to participants: (i) *healthcare professionals involved* and (ii) *family support.*

Regarding “healthcare professionals involved,” this subtheme encompasses participants’ perceptions of which healthcare professionals are engaged in the oncological treatment process and how they can provide assistance through their professional roles. Within this context, the physician was unanimously identified as the primary professional associated with cancer care, followed by the dentist, psychologist, nutritionist, and nurse. The physiotherapist was mentioned by only one participant. Participants expressed trust and hope in the conduct of the professionals involved, and one emphasized the importance of receiving clear and coherent information from healthcare providers following a cancer diagnosis.

The subtheme “family support” addresses information related to the assistance offered by family members. All participants identified their families as a source of support, particularly in attending medical appointments and examinations, understanding their health conditions, and providing both emotional and physical support throughout the oncological process.

Table 5 presents representative excerpts for each subtheme that emerged from the main theme 4.

**Table 5:** Third-party support and its subthemes

Theme	Subtheme	Representative excerpt
Third-party support	Healthcare professionals involved	**A.S., male:** “Those therapists, doctors, especially, and… I don’t know… others I don't know (laughs).”
		**L.F.C., male:** “The nutritionist’s part will be very helpful, because there will be phases where you won’t be able to manage with a normal diet […] also the psychological part, because your mind has been racing, really! […] And the dentist, who will be doing laser treatment on your mouth. […] I don't know (makes a doubtful expression, furrowing her brow) if there are any more.”
		**N.D., male:** “Endocrinologist, the oncologist part. […] So to tell you the truth, it’s a lot for so few people, you know? There’s nothing more you could want, right? As they say, the only thing more there is is God, so that’s it. I think we don’t need any more professionals, right…”
	*Family support*	**M.V., male:** “I’m feeling very strong, and my family is supporting me too, right?!”
		**R.C.N., female:** “My sister goes there with me. They haven’t abandoned me for a single day, thank God she stayed with me the whole time at the hospital, during surgery, after surgery, and she’s been supporting me. She said, ‘I’ll only stop when you start chemo and you see that you’re doing well, then you can manage as best you can…’”
		**J.A.S., male:** “I go to several doctors and each one says something different. Since I’m practically illiterate, I can’t understand anything, I forget what they say… But my daughter remembers everything!”

### Theme 5: context of the diagnosis

In this theme, two subthemes emerged that constitute the context of diagnosis: (i) *impact of diagnosis* and (ii) *process for obtaining a diagnosis*.

The subtheme “impact of diagnosis” addresses how receiving a cancer diagnosis affected participants in physical, emotional, social, and occupational aspects. Participants also described changes in their daily routines and those of their families as a result of the diagnosis and its related symptoms. All participants reported that the diagnosis had some degree of impact on their lives, leading to modifications in personal or family routines.

Regarding the subtheme “process for obtaining a diagnosis,” participants described the difficulties encountered in receiving an accurate cancer diagnosis. These included initial misdiagnoses prior to the correct identification of cancer, as well as delays in receiving confirmation and in initiating prescribed oncological treatment. Six participants reported that their diagnoses were delayed due to previous medical errors, and one participant highlighted that “sometimes the population does not have all that time to wait.”

Table 6 presents representative excerpts for each subtheme that emerged from the main theme 5.

**Table 6:** Context of the diagnosis and its subthemes

Theme	Subtheme	Representative excerpt
Context of the diagnosis	Impact of diagnosis	**L.F.C., male:** “Today it’s affecting my family more, you know. My parents, siblings, uncles. Yeah, I’m trying to figure out how to talk to my children about it.”
		**C.O.P., male:** “Ah, because we’re used to working. Then I’ve already been away from work, you know. So I go to my sister’s house, and I live on a farm. Sometimes I go to my son’s house. So a lot of things change, if you have to go back and forth, you're not at home.”
		**R.C.N., female:** “No, I’m not doing anything, am I? I spend most of my time walking around the house, doing nothing! That’s my routine, doing nothing! I used to do laundry, iron, cook, teach, I sold costume jewelry, I had a hectic life, my life was crazy. I had three jobs. I handled it all perfectly… I never had a sedentary life, not at all!”
		**S.B.S., male:** “So, before the [biopsy] results came out, she [my wife] was suffering from very severe depression… She threw alcohol on herself and set herself on fire. She burned to death. Then I got a little discouraged, afterwards… And because of them, her (points to his daughter), her sister, my grandson, I cheered up. I said, “No, I’m going to run, this isn’t how it ends.” It was really bad. It still is, you know? She was my companion. Instead of her being here (points to his companion), her mother should be… always with me.”
	Process for obtaining a diagnosis	**N.G.S., female:** "She had a lot of tests done, and they said it was an arrhythmia, and she went to the doctor, arrhythmia… she started coughing in March, a cough, cough, cough that wouldn’t go away, so I went to a lung specialist… then he did tests and found a lesion in her lung, she only found out through a CT scan, then the doctor said it’s [cancer]… she did a biopsy and everything.”
		**M.J.R., female:** “I discovered this lump (shows where on her neck), this lump has been bothering me since last year. I was only getting ultrasounds, then I got more worried and went to the doctor [surgeon], that's when he said it would be good to remove it to do a biopsy and that's how they found out.”
		**J.P.V., male:** “But the doctor kept saying it was a heart arrhythmia… My heart was enlarged. I went back to him months later and said, ‘I’m going to get tested,’ and then my heart was normal. I said, ‘You [the doctor] didn’t say it was an arrhythmia, where is it?’ Then he said to have a lung exam, a test… Then he did it and discovered [the cancer].”
		**L.F.C., male:** “Sometimes the population doesn’t have all that time, a year and a half, two years, to get that diagnosis. So, the population is lacking, especially the less informed people who don’t know where to look for help…”

## Discussion

The findings of this qualitative study demonstrated that individuals diagnosed with lung or head and neck cancer, prior to initiating oncological treatment, exhibited a low level of knowledge regarding the disease, their prescribed oncological therapies, potential symptoms, and the role of physical exercise as an adjunctive intervention during treatment. Furthermore, participants expressed uncertainty and insecurity about the feasibility of engaging in physical exercise while diagnosed with cancer and during oncological therapy. Nevertheless, they showed willingness to participate if exercise programs were proposed as part of their treatment process.

To date, no studies have specifically investigated the level of knowledge of individuals with cancer regarding their disease, prescribed oncological treatments, and exercise prior to the start of therapy. Existing studies mainly address perceptions related to oncological treatment and physical exercise after these interventions have been implemented.

Concerning knowledge of disease, a qualitative evidence synthesis conducted among individuals at high risk for colorectal cancer revealed a similar theme—“limited knowledge of colorectal cancer and its risk factors.” The authors concluded that healthcare professionals could play a central role in promoting healthy lifestyle habits, considering the individual’s knowledge and social support [24]. In another study conducted in Portugal, investigating perceptions and attitudes toward lifestyle-related risk factors for prostate cancer, family, friends, and healthcare professionals were identified as major motivators for behavior change, driven by the fear of cancer consequences and the desire for increased survival. However, barriers to lifestyle modification also emerged, including limitations imposed by the disease itself [25], corroborating the subtheme “availability to perform physical exercise” observed in the present study. Understanding modifiable risk factors, disease-related knowledge, and how individuals process health information is essential for developing effective health education strategies that support early cancer detection and screening, particularly for lung and head and neck cancers, where such initiatives remain limited.

Regarding knowledge of physical exercise, a descriptive study investigating awareness and perceived benefits of exercise among women who had completed primary treatment for breast cancer found that only 22% of participants were familiar with recommended exercises for this population. The main sources of information were television (24%) and the internet (19%). Among those who were aware, 52% understood that exercise should include resistance, flexibility, and aerobic training performed three to five times per week, while 94% recognized the benefits of exercise [26].

Consistent with these findings, the present study also revealed a low level of knowledge regarding physical exercise, particularly in relation to feasible modalities during oncological treatment and the perceived impact of physical debilitation. Although breast cancer is highly prevalent worldwide, with well-established screening campaigns, there remains a lack of education and counseling from healthcare professionals regarding the role of exercise before, during, and after cancer treatment [27, 28].

A meta-synthesis involving patients with various cancer types undergoing chemotherapy explored perceptions of both the treatment and exercise as an adjunctive therapy. Emergent subthemes included (i) “physical side effects impacting daily life”; (ii) “deterioration of physical capacity”—which aligns with the present study’s subtheme “impact of symptoms”; (iii) “positive benefits despite side effects”; and (iv) “relief of anxiety, low mood, and depressive feelings,” consistent with the subtheme “effects of physical exercise.” Additionally, subthemes such as “professional supervision and advice” and “social and family support” also align with the present theme “third-party support” [18].

Patients diagnosed with cancer often experience a strong sense of helplessness related to physical deterioration imposed by the disease and its treatments, particularly chemotherapy. However, after engaging in exercise-based interventions, they report physical and psychological benefits, perceiving exercise as a strategy to cope with emotional distress [18]. Notably, in the present study, this feeling of helplessness was observed even before the initiation of cancer treatment, underscoring the need for healthcare professionals—including physiotherapists, nurses, and physicians—to provide reassurance and emotional support from hospital to outpatient settings. Clear communication about exercise as a means to mitigate physical and psychological burden may enhance patients’ sense of control and emotional well-being [18].

Chang et al. [16] conducted a qualitative study exploring exercise experiences among patients with metastatic lung cancer, whose demographic profile closely resembled the current sample (predominantly male, married, ECOG 1–2). Emergent themes included (i) “modifying exercise to maximize physical function,” consistent with the subtheme “what the exercises would be like,” in which participants expressed the need for adaptations; (ii) “living with symptoms and frustrations but continuing to exercise,” reflecting the subtheme “availability to perform physical exercise,” where participants stated they would exercise “to heal” or “to improve” within their limitations; and (iii) “exercising to sustain hope, inner strength, and life,” indicating that exercise fosters hope for survival [29].

A qualitative study involving patients with lung or head and neck cancer identified barriers and facilitators to rehabilitation access from the perspective of healthcare professionals. Among seven emergent themes, four specifically addressed barriers to exercise-based rehabilitation programs: (i) “understanding patient resources,” (ii) “collaboration determining access,” (iii) “education, knowledge, and evidence influencing access,” and (iv) “resource availability affecting rehabilitation services” [30]. These themes highlight financial constraints, limited rehabilitation centers, and insufficient access to specialized oncological rehabilitation services. Education and knowledge reemerged as critical factors, reinforcing the global need for strategies to mitigate public unawareness regarding lung and head and neck cancers, their treatments, symptoms, and exercise as a therapeutic option. Such factors directly influence access to diagnosis, timely treatment, and overall physical, mental, and social outcomes, potentially reducing healthcare costs.

To evaluate the effectiveness of educational interventions, a study conducted among healthy university women compared breast cancer knowledge before and 15 days after an educational program. The intervention consisted of 45–60-min sessions in dormitories, with groups of 10–15 participants, using pamphlets, lectures, and discussions. At the end of each session, participants were encouraged to summarize their understanding, ensuring comprehension. The study demonstrated a significant increase in breast cancer knowledge (*p* < 0.001) and treatment awareness (*p* < 0.001) after the intervention [28].

It is widely recognized that individuals with cancer, as well as cancer survivors, will experience some degree of impairment in physical performance as a result of adverse effects associated with oncological treatments. In this context, rehabilitation and prehabilitation interventions are essential to minimize functional impairments and preserve physical capacity throughout the entire continuum of cancer care [7, 31]. Therefore, one of the most effective strategies for incorporating physical exercise into oncology clinical practice is health education, beginning with healthcare professionals themselves. Within this context, physiotherapists play a central role in promoting awareness of the benefits of physical exercise before, during, and after cancer treatment, supported by robust scientific evidence, including well-established international guidelines and recommendations, particularly for individuals with lung cancer and head and neck cancer [7, 12, 32, 33].

However, despite well-established recommendations supporting the integration of physical exercise into cancer care, prehabilitation and rehabilitation services remain relatively underutilized in many countries, representing an important gap in oncology care. A moderate proportion of thoracic surgeons recognize the need to refer patients for prehabilitation before undergoing lung resection [34]. Nevertheless, 33% of surgeons reported being uncertain about which healthcare professional should receive referrals for exercise-based interventions as an adjunctive treatment, thereby reducing referral rates for structured exercise programs [34]. This reality contributes to the growing burden of cancer-related morbidity, especially considering that the number of cancer survivors is expected to nearly double over the coming decades [35].

In addition, sedentary behavior among individuals with cancer and cancer survivors is influenced not only by physical barriers, such as disease- and treatment-related symptoms, but also by limiting beliefs that they are incapable of engaging in any form of physical exercise due to their current health condition [36, 37]. Furthermore, professional barriers, including a lack of confidence among healthcare providers and a shortage of qualified professionals to prescribe and supervise exercise interventions, have been consistently reported. Additional challenges related to access to cancer exercise programs, such as geographical distance, transportation difficulties, and financial constraints, are also well documented [9, 38, 39].

Therefore, health education represents a key strategy for promoting behavior change, enhancing patient activation in cancer care, and facilitating the effective integration of exercise as a component of oncology treatment [38, 39]. Nevertheless, important gaps within healthcare systems continue to contribute to educational deficits, including the limited dissemination of accurate, high-quality information and the frequent presentation of information in formats that are not easily understandable for the target population [38, 40].

Particularly among patients with cancer, health literacy significantly influences disease understanding and affects the ability to comprehend and apply treatment-related information in practice [38, 40]. Consequently, this challenge is further intensified when information is not adapted to the patient’s level of understanding, leading to reduced adherence to treatment recommendations and self-management strategies, ultimately limiting patient activation and engagement in cancer care [39]. Therefore, tailoring health information to the population’s literacy level and utilizing accessible dissemination tools, such as social media platforms and primary healthcare outpatient settings, are essential for empowering patients and promoting the dissemination of appropriate information, thereby improving understanding of and adherence to available oncological treatments.

### Study limitations and future directions

Although this knowledge gap has not been previously investigated, further research involving other cancer types is warranted to ensure broader representation and generalizability of findings across the cancer population. The structure of the healthcare system in Brazil should also be taken into consideration, as it differs from healthcare systems in other countries. Nevertheless, we believe that limited patient knowledge regarding cancer and its management is likely a common issue worldwide.

Additionally, participants’ educational attainment should be considered when interpreting the findings. Future studies should employ validated instruments to assess educational level and health literacy, thereby reducing the potential influence of cognitive and comprehension-related biases and improving the accuracy of the results.

## Conclusion

It can be concluded that individuals diagnosed with lung or head and neck cancer, prior to beginning oncological treatment, exhibit a low level of knowledge regarding the disease, prescribed therapies, expected symptoms, and the role of physical exercise during treatment. Participants also demonstrated limited or no understanding of what such exercise programs might involve, yet expressed willingness to engage in them if proposed.

## Supplementary Information

Below is the link to the electronic supplementary material.

Supplementary file1 (PDF 339 kb)

Supplementary file2 (PDF 474 kb)

## Data Availability

The data supporting the findings of this study are not publicly available due to confidentiality restrictions but may be obtained from the corresponding author upon reasonable request. The data are stored in a controlled-access repository at the Laboratory of Mucus-Secreting System Studies, Faculty of Science and Technology (FCT/UNESP).
