# Supplementary material for: “No… Nobody told me anything about that”: a qualitative study on pretreatment knowledge of individuals with lung or head and neck cancer regarding the disease, treatments, and physical exercise
Source: Support Care Cancer. 2026 Oct 27;34(11):1158. doi: 10.1007/s00520-026-11407-1 (PMC13710653; doi:10.1007/s00520-026-11407-1)
Supplement: Supplementary file 1 [file 520_2026_11407_MOESM1_ESM.pdf]

Journal name: Supportive Care in Cancer

“No… Nobody Told Me Anything About That”: A qualitative study on pretreatment knowledge of individuals with lung or head and neck cancer regarding the disease, treatments, and physical exercise

Isabela Duarte a

Karina Arielle da Silva Souza a

Alice Haniuda Moliterno a

Paloma Borges a

Matheus Pedroso a

Isis Grigoletto b,c

Ercy Mara Cipulo Ramos a

List of affiliations

a. Department of Physiotherapy, São Paulo State University, School of Technology & Science (FCT/UNESP), Presidente Prudente, Brazil.
b. Curtin School of Allied Health, Curtin University, Perth, WA, Australia.
c. Curtin School of Nursing, Curtin University, Perth, WA, Australia.

E-mail address of the corresponding author: isabela.duarte@unesp.br

Semi-structured interview guide

This supplementary material presents the semi-structured interview guide applied to the study participants. It comprises ten open-ended questions addressing knowledge about cancer, the treatments to be undertaken, the symptoms to be experienced, and the role of physical exercise as an adjuvant to cancer treatment.

1. What do you know about cancer?
2. How do you believe cancer treatment is conducted?
3. What do you believe is important for you to receive high-quality treatment?
4. Which symptoms do you think cancer and its treatment may cause? Have you already experienced any of these symptoms?
5. Do you believe these symptoms may interfere with your life? In what way?
6. In your opinion, which healthcare professionals can assist in your treatment? How do you believe they can help you?
7. What do you know about physical exercise in patients with cancer?
8. Do you believe physical exercise can be performed during cancer treatment? Why?
9. In what way do you believe physical exercise can influence the symptoms caused by cancer and its treatment?
10. Based on everything discussed, would you like to add any additional information regarding your cancer treatment?
