# Supplementary material for: “No… Nobody told me anything about that”: a qualitative study on pretreatment knowledge of individuals with lung or head and neck cancer regarding the disease, treatments, and physical exercise
Source: Support Care Cancer. 2026 Oct 27;34(11):1158. doi: 10.1007/s00520-026-11407-1 (PMC13710653; doi:10.1007/s00520-026-11407-1)
Supplement: Supplementary file 2 [file 520_2026_11407_MOESM2_ESM.pdf]

Journal name: Supportive Care in Cancer

“No… Nobody Told Me Anything About That”: A qualitative study on pretreatment knowledge of individuals with lung or head and neck cancer regarding the disease, treatments, and physical exercise

Isabela Duarte [a]

Karina Arielle da Silva Souza [a]

Alice Haniuda Moliterno [a]

Paloma Borges [a]

Matheus Pedroso [a]

Isis Grigoletto [b,c]

Ercy Mara Cipulo Ramos [a]

List of affiliations

a. Department of Physiotherapy, São Paulo State University, School of Technology & Science (FCT/UNESP), Presidente Prudente, Brazil.
b. Curtin School of Allied Health, Curtin University, Perth, WA, Australia.
c. Curtin School of Nursing, Curtin University, Perth, WA, Australia.

E-mail address of the corresponding author: isabela.duarte@unesp.br

Thematic analysis

This supplementary material presents the thematic analysis, including all representative excerpts for each theme and subtheme identified in the interviews.

Transcript	Participant	Theme	Subtheme	Representative excerpt
1 (pg 1)	P1	General knowledge	Knowledge about the disease	**P1:** "I know, it's a malignant disease, right? And it's very difficult, you have to have a lot of faith in God, patience, right? To overcome it, right?"
1 (pg1)	P1	General knowledge	Knowledge about the disease	**P1:** “[...] because I can’t even explain what that is in my life, I don’t know... It’s difficult...”
2 (pg 1)	P2	General knowledge	Knowledge about the disease	**P2:** "Ah... died (a laugh along with a shake of the head in denial)."
3 (pg 1)	P3	General knowledge	Knowledge about the disease	**P3:** "It's a cell that ends up being aggressive to the body, and you never expect to have cancer at thirty-six years old, actually..."
4 (pg 1)	P4	General knowledge	Knowledge about the disease	**P4:** "I took care of my two brothers who had cancer, and I

				know it's not a good experience at all."
5 (pg 1)	P5	General knowledge	Knowledge about the disease	**P5:** "Because it's one of the worst [diseases] there is, isn't it?"
6 (pg 1)	P6	General knowledge	Knowledge about the disease	**P6:** "Oh, I don't know anything, honey, I just know, you know, it's not a good type, right? Now, what kind of cancer I have has to do with my daughter who came with me."
6 (pg 1)	P6	General knowledge	Knowledge about the disease	**P6:** "It can be moved from one place to another, right?"
7 (pg 1)	P7	General knowledge	Knowledge about the disease	**P7:** "I know they're dead cells, right? They die, they clump together, they settle down, and that's what happens, that's what I know... they're tumors that sometimes a person can't even tell about."
7 (pg 2)	P7	General knowledge	Knowledge about the disease	**P7:** "When this happened to my son... It was one of the most aggressive cancers the doctor said he could have [...] that's when I went to see, to learn, that it's an aggressive disease, that there are several types of cancer, several types of tumors... an infinite number of things that one can aggravate or affect or develop, that everyone has in their blood, right..."
8 (pg 1)	P8	General knowledge	Knowledge about the disease	**P8:** "Oh honey, I can't even say what I know, I only found out after June 10th."
9 (pg 2)	P9	General knowledge	Knowledge about the disease	**P9:** "It's a complicated thing, the first impression is of an incurable disease. It fights… How do you say it? Red blood cells, things like that, causing a deficiency, and then the end result was the same, right (light laughter), like the end result for everyone, whether you have it [cancer] or not. My daughter, we all go to the same place."
10 (pg 1)	P10	General knowledge	Knowledge about the disease	**P10:** "I don't know anything!"
11 (pg1)	P11	General knowledge	Knowledge about the disease	**P11:** "Ah, I think it [cancer] is a disease, like mine, it's a very aggressive disease and it ends

				up destroying a person's health, it consumes their life, that's what I think."
12 (pg 1)	P12	General knowledge	Knowledge about the disease	**P12:** "Faith, because this disease is rare, very rare indeed, and I am aware of these things. So, I see, I'm always watching television, right? So, we're keeping up with it, it's not easy..."
7 (pg 2)	P7	General knowledge	Knowledge about the disease	**P7:** "So then, you start thinking, 'This won't happen to me,' but when it knocks on your door, that's when you want to understand what it is, and then it's too late, there's nothing left to understand, it's right there, you have to treat it... I don't think I ever stopped to think about cancer."
2 (pg 6)	P2	General knowledge	Knowledge about the disease	**P2:** "I used to smoke, a lot [...] I quit two months ago [...] It might have interfered, but 60 years later? It could have interfered 50 years ago (gesturing with his hand simulating the past)."
3 (pg 4)	P3	General knowledge	Knowledge about the disease	**P3:** "For every hundred million, there's one person, and where did that percentage come from for me? So, we don't know where it came from, why it happened, the consideration of vaccination that was discussed these days? It happened! But we don't know what actually changed that cell, do we?"
6 (pg 2)	P6	General knowledge	Knowledge about the disease	**I.C.D.A.:** "And can you imagine what things happen in life and some habits that can lead to [cancer] appearing?"<br>**P6:** "No."
7 (pg 2)	P7	General knowledge	Knowledge about the disease	**P7:** "It only takes developing something like that, which leads to... developing this [cancer]. I don't know if it's because I smoked, but it's been over 20 years since I quit, but I think I'm still paying for it."
8 (pg 1)	P8	General knowledge	Knowledge about the disease	**P8:** "But I never expected I would get [cancer], but it's genetic, it runs in the family."

6 (pg 2)	P6	General knowledge	Knowledge about the disease	**P6:** "Because my case is in my lung, right? And there's already a little bit in my spine and a little bit in my brain... a little spot, you know?"
7 (pg 1)	P7	General knowledge	Knowledge about the disease	**P7:** I looked at the doctor and asked, "Is it malignant?" He said, "Yes..." and now he's said, "We'll have to operate," and I said, "Yes sir, that's what I'm here for!"
7 (pg 1)	P7	General knowledge	Knowledge about the disease	**P7:** "But then he [the doctor] came and said, 'But you won't escape chemotherapy and/or radiotherapy, because we're not sure if anything from this surgery was lost,' that's when I got scared, I was terrified, I started crying a lot…"
6 (pg 1)	P6	General knowledge	Knowledge about the disease	**P6:** "Ah... I believe in miracles, because I have a nephew who had a brain injury... It's been five years since he died, he's in Japan now, he recovered... my nephew was a miracle."
7 (pg 1)	P7	General knowledge	Knowledge about the disease	**P7:** "Oh, I know that cancer is a terrible disease, I lost my son last year to liver cancer."
7 (pg 1)	P7	General knowledge	Knowledge about the disease	**P7:** "We've had several cases in the family, so we live on high alert, you know?"
8 (pg 1)	P8	General knowledge	Knowledge about the disease	**P8:** "My father died of cancer, my sister, my niece, and two of my brothers also have it. They were the only ones who received treatment, thank God. They were treated here five years ago, but they're doing well, they're cured, thank God."
10 (pg 1)	P10	General knowledge	Knowledge about the disease	**P10:** "I've already lost an aunt. I've already lost a cousin... all to these diseases. So, what I can say is that it [the disease] is tragic, isn't it?"
2 (pg 4)	P2	General knowledge	Understanding cancer treatment and symptoms	**P2:** It's because if... the patient has never done it, how will he know? It's like going fishing, you have to dig up worms. If you've never dug up worms, how are you going to fish?

2 (pg 4)	P2	General knowledge	Understanding cancer treatment and symptoms	**I.C.D.A:** "Have you ever spoken to anyone who has undergone this type of treatment?" **P2:** "No, I never, I never imagined I would get sick. Maybe what's good for me isn't good for others."
3 (pg 1)		General knowledge	Understanding cancer treatment and symptoms	**P3:** "I've never been like that, I've never pulled so much stuff from the internet... because sometimes it might not be useful for me, so I prefer to live more in the moment, actually."
5 (pg 1)	P5	General knowledge	Understanding cancer treatment and symptoms	**P5:** "I've never talked to anyone who's actually done anything like that, you know."
1 (pg 5)	P1	General knowledge	Understanding cancer treatment and symptoms	**P1:** "It's going to burn, isn't it? (puts hand to throat), I'm going to feel really nauseous, right?"
2 (pg 4)	P2	General knowledge	Understanding cancer treatment and symptoms	**P2:** "Cancer, all [the symptoms]."
2 (pg 4)	P2	General knowledge	Understanding cancer treatment and symptoms	**P2:** "Evil feels bad, and from then on it gets worse and worse... (shaking head negatively)."
3 (pg 2)	P3	General knowledge	Understanding cancer treatment and symptoms	**P3:** "They already said it will probably cause a mouth ulcer, I'll just have soup instead, which I don't really like."
3 (pg 2)	P3	General knowledge	Understanding cancer treatment and symptoms	**P3:** "Well... here comes the funny part, where I'll probably go bald."
3 (pg 3)	P3	General knowledge	Understanding cancer treatment and symptoms	**P3:** "Oh, it's going to happen, will I become debilitated? Maybe I will, maybe I won't. Will I go bald? Maybe I will, maybe I won't. So, suffering in advance, I think that's worse."
4 (pg 3)		General knowledge	Understanding cancer treatment and symptoms	**P4:** "Well, something else I've learned is that we don't have an appetite, how do you say it, we don't taste anything."
5 (pg 1)	P5	General knowledge	Understanding cancer treatment and symptoms	**P5:** "Then he [the doctor] said, 'The cough might get a little worse, it's like an inflammation, I don't know what. [...] You feel pain from time to time, you know.'"
5 (pg 2)	P5	General knowledge	Understanding cancer treatment and symptoms	**P5:** "Well, I don't know, hopefully it wouldn't cause so much damage (he smiles

				shyly). Some people say, when they find out, 'oh, therapy is a bit harsh,' and so on."
6 (pg 3)	P6	General knowledge	Understanding cancer treatment and symptoms	**P6:** "The doctor (she) said it can cause diarrhea, it can cause vomiting, right? It depends on each case, right? That's what she told me..."
7 (pg 2)	P7	General knowledge	Understanding cancer treatment and symptoms	**P7:** "I'm afraid of chemotherapy, it's not the fear of going bald, it's nothing like that… it's the fear of the reactions, you know? Because some people get sick, some people say it's horrible, that they vomit, that they lose weight, my fear of chemo is getting sick, sicker than I got [before the surgery]."
7 (pg 3)	P7	General knowledge	Understanding cancer treatment and symptoms	**P7:** "I have no idea, I have no idea what chemotherapy symptoms are, I don't know doctor, I can't tell you because I've never had it, I've never seen it, so I don't know what it is."
9 (pg 6)	P9	General knowledge	Understanding cancer treatment and symptoms	**P9:** "Look, as far as I know, it can cause some problems, specifically digestive issues, headaches, and some precautions we need to take regarding cold, heat, and low immunity; we have to avoid that sort of thing."
10 (pg 3)	P10	General knowledge	Understanding cancer treatment and symptoms	**P10:** "I hope nothing happens, that I don't have any symptoms."
12 (pg 2)	P12	General knowledge	Understanding cancer treatment and symptoms	**P12:** "It's going to affect our bodies, right? I know it's going to shake things up. It's not like it's going to happen and then everything will be normal. When the treatment starts, it's going to affect our bodies [...] It's weakness, right? Weakness!"
14 (pg 3)	P14	General knowledge	Understanding cancer treatment and symptoms	**P14:** "Now, there's a risk there, right, that the chemo and radiation could... could cause swelling and try to block your trachea. Then they'll have to intervene, they'll put in a tube to interfere, right? But he said that maybe it won't even happen. It's only a last resort."

3 (pg 2)	P3	General knowledge	Understanding cancer treatment and symptoms	**P3:** "Ah, in principle, we know two things: it's going to be injectable, right?"
4 (pg 1)	P4	General knowledge	Understanding cancer treatment and symptoms	**I.C.D.A.:** "No one ever said anything to you about how it's going to be, what you're going to do?"<br>**P4:** "No, no. My colleague who had radiotherapy said she went into a machine."
6 (pg 3)	P6	General knowledge	Understanding cancer treatment and symptoms	**P6:** "Ah... It's chemotherapy, right? But I don't know how it works, which I don't think is good, is it?"
7 (pg 2)	P7	General knowledge	Understanding cancer treatment and symptoms	**P7**: "I have no idea, because I've never seen it done, nor do I know what it's like. I know there's injectable chemo, there's chemo that's... in pill form, liquid, but I've never seen anyone take chemo, I've never seen anyone undergo chemotherapy treatment, so I don't know, I don't know what it's like..."
8 (pg 2)	P8	General knowledge	Understanding cancer treatment and symptoms	**I.C.D.A.:** "And do you know what radiotherapy and chemotherapy are like?"<br>**P8:** "No, I've never seen it, daughter. I've never seen it. I can't even explain to you what it's going to be like, because I've never seen it, you know? Only when I see it..."
9 (pg 2)	P9	General knowledge	Understanding cancer treatment and symptoms	**P9:** "I'm the kind of person who doesn't worry too much about knowing details."
11 (pg1)	P11	General knowledge	Understanding cancer treatment and symptoms	**P11:** "I'll have to wear some masks, some kind of thing, to protect myself and avoid burning my skin. They say it's radiation, so I can only really know for sure the day I go through it..."
13 (pg 2)	P13	General knowledge	Understanding cancer treatment and symptoms	**P13:** "My daughter is a pharmacist, but I've never heard of chemotherapy."
14 (pg 2)	P14	General knowledge	Understanding cancer treatment and symptoms	**P14:** "The doctor (he) said that after we finish the tests we're doing now, there's one last one to do. He said it will basically be alternating radiation and chemotherapy. The two. And

				if that doesn't work, then it would be a surgical intervention. But I think it will work out."
1 (pg 8)	P1	General knowledge	Performing physical exercise during cancer treatment	**I.C.D.A.:** What do you know about physical exercise in patients diagnosed with cancer?<br>**P1:** "I don't know. I know nothing, I'm a layman."
2 (pg 13)	P2	General knowledge	Performing physical exercise during cancer treatment	**I.C.D.A.:** "And would you like, sir, to know more about that, about the exercise?"<br>**P2:** Yes, I would love to!
2 (pg 13)	P2	General knowledge	Performing physical exercise during cancer treatment	**I.C.D.A.:** "And do you think it would be important for us to explain at the beginning what the exercise would entail?"<br>**P2:** Yes, that would be very important!
3 (pg 9)	P3	General knowledge	Performing physical exercise during cancer treatment	**P3:** "I haven't heard of it because my research is very limited. And besides, to what extent will this patient be debilitated?"
4 (pg 4)	P4	General knowledge	Performing physical exercise during cancer treatment	**P4:** "Ah, I've heard of it before."
6 (pg 5)	P6	General knowledge	Performing physical exercise during cancer treatment	**I.C.D.A.:** "And you, ma'am, have you ever heard of people who have cancer and exercise while having cancer, while undergoing cancer treatment?"<br>**P6:** "No (surprised tone)!!!"
7 (pg 5)	P7	General knowledge	Performing physical exercise during cancer treatment	**P7:** "I've heard that at the hospital they made me exercise while I was there."
8 (pg 6)	P8	General knowledge	Performing physical exercise during cancer treatment	**P8:** "Ah, I can't even speak, because I've never heard of it, right? If I ever had that perspective, I could even give an answer, but I've never heard of it."
11 (pg 5)	P11	General knowledge	Performing physical exercise during cancer treatment	**P11:** "No... Nobody told me anything about that."
1 (pg 3)	P1	Expectations during cancer treatment	What to expect from cancer treatment	**P1:** "Look, from what she explained to me, it won't be that difficult, right?"
1 (pg 4)	P1	Expectations during cancer treatment	What to expect from cancer treatment	**P1:** "Because I'm going to have a lot of willpower, right? You have to have strength, hope, right? And not give up!"

1 (pg 5)	P1	Expectations during cancer treatment	What to expect from cancer treatment	**P1:** "Ah, the beginning is going to be kind of difficult, right?"
2 (pg2)	P2	Expectations during cancer treatment	What to expect from cancer treatment	**P2:** "Yeah... we're going to feel bad [...] we won't be able to walk... Yeah, we won't be able to do anything with our lives. All our thoughts are negative."
2 (pg2)	P2	Expectations during cancer treatment	What to expect from cancer treatment	**I.C.D.A.:** "And you, sir, think this treatment doesn't produce a benefit?"<br>**P2:** "Mine, I still don't... I don't know, honestly, I don't know. Apparently, I don't think so..."
3 (pg 1)	P3	Expectations during cancer treatment	What to expect from cancer treatment	**P3:** "It's a phase that will also take a toll on my body, because we already know a little about the effects."
9 (pg 1)	P9	Expectations during cancer treatment	What to expect from cancer treatment	**P9:** "So, at first I thought it would be a little more complicated, but I actually found it to be quite simple. Considering how serious I thought it was... I don't know if I'm even underestimating the disease, you know? But for me it's fine. I'm not someone who worries too much."
9 (pg 2)	P9	Expectations during cancer treatment	What to expect from cancer treatment	**P9:** "That's why, sometimes, with all the resources we have today, we don't worry too much, do we?"
11 (pg 1)	P11	Expectations during cancer treatment	What to expect from cancer treatment	**P11:** "Ah, I think my treatment, I think it's going to be very good!"
12 (pg 1)	P12	Expectations during cancer treatment	What to expect from cancer treatment	**P12:** "If it doesn't always cure it [cancer], sometimes it provides some relief... So, it's a little relieving for us, right..."
1 (pg 5)	P1	Expectations during cancer treatment	What to expect from healthcare professionals	**P1:** "So you are capable. I believe in you too. I believe in God and in you! May He guide you so that everything goes well!"
4 (pg 4)	P4	Expectations during cancer treatment	What to expect from healthcare professionals	**I.C.D.A.:** "What do you, ma'am, expect from them [healthcare professionals]?"<br>**P4:** "Ah, I expect everything. But I don't know what 'no' means."
7 (pg 1)	P7	Expectations during cancer treatment	What to expect from healthcare professionals	**P7:** "I have complete faith in the doctor and in God (strong affirmation), I am certain that

				with their help and yours, I will get through this."
1 (pg 9)	P1	Expectations during cancer treatment	What to expect from healthcare professionals	**P1:** "So you guys aren't going to do something that's useless. So, I believe that whatever you do is good! (laughs)"
2 (pg 3)	P2	Expectations during cancer treatment	What to expect from healthcare professionals	**I.C.D.A.:** "And the physiotherapist, why do you think they're important?"<br>**P2:** Ah, we need to breathe, right?
6 (pg 5)	P6	Expectations during cancer treatment	What to expect from healthcare professionals	**P6:** "Watching so I don't get sad, right? Not letting myself go, without contact with people, right? Giving me strength, I can't get discouraged [psychologist]."
7 (pg 8)	P8	Expectations during cancer treatment	What to expect from healthcare professionals	**P8:** "As a physiotherapist, if you tell me, 'I'm going to give you this type of exercise that will help you recover from chemo and make you feel less unwell,' I'll do it, you understand?"
11 (pg 2)	P11	Expectations during cancer treatment	What to expect from healthcare professionals	**P11:** "The speech therapist, the way she's going to help me is to be able to speak normally, the way I used to talk before. With the nutritionist, she'll... but what about... how do you put it? About basic nutrition, so I don't lose weight, to maintain what I was... I believe she'll do that. She's doing that for me."
5 (pg 3)	P5	Expectations during cancer treatment	What to expect from healthcare professionals	**P5:** "It's like in the case I was talking about. It's been good everywhere I go... so many attendants at the front, so many nurses who attend inside, and doctors too."
7 (pg 3)	P7	Expectations during cancer treatment	What to expect from healthcare professionals	**P7:** "But since I started treatment after the surgery, I've been very well taken care of by everyone, thank God, so I have nothing to complain about."
3 (pg 3)	P3	Expectations during cancer treatment	What to expect from healthcare professionals	**P3:** "Being consistent with the patient, don't leave out any information, say things like 'we're doing this because of this and that,' 'the probability of a cure is this.'"

1 (pg 5)	P1	Expectations during cancer treatment	What to expect from healthcare professionals	**P1:** “[...] the problem is just talking, right? (puts hand to throat). Yeah... it's become difficult to talk (passes hand over neck).”
1 (pg 5)	P1	Expectations during cancer treatment	What to expect from healthcare professionals	**P1:** "Yeah... this tumor came out here (puts hand to neck) and it's closing my throat."
2 (pg 5)	P2	Expectations during cancer treatment	What to expect from healthcare professionals	**P2:** "I don't feel any pain. My only problem is (simulates breathing and coughing)... breathing and walking. I'm going from here to the corner, I'm already tired there, and if I go back without holding onto anything, I'm (makes gestures with his head simulating as if he were lost) I'm lost."
3 (pg 4)	P3	Expectations during cancer treatment	Impact of symptoms	**P3:** "I feel spasms and I've been complaining for a few days now about headaches [...] difficulty breathing, because it's compressing my sinuses a bit [...] and some hearing loss, but that's because it's coming and compressing my sinuses."
5 (pg 2)	P5	Expectations during cancer treatment	Impact of symptoms	**P5:** "I have coughing fits, so when they come on, there's no stopping them. Oh, the rest of my body is fine."
6 (pg 4)	P6	Expectations during cancer treatment	Impact of symptoms	**P6:** "I feel unwell, and my legs are kind of weak, you know? All day long... I feel fine sleeping, lying down like this I feel good, but even just walking around the house I'm trembling."
6 (pg 4)	P6	Expectations during cancer treatment	Impact of symptoms	**P6:** "I have back pain, but I think it's from my lung [where the cancer is], right?"
7 (pg 4)	P7	Expectations during cancer treatment	Impact of symptoms	**P7:** "The only thing I had before the surgery was horrible pain. [...] I couldn't eat because of the pain... the pain was so intense that all I could do was cry and take painkillers. I wasn't hungry, I didn't want to eat, not because I didn't want to eat, but because of the pain."
8 (pg 4)	P8	Expectations during cancer treatment	Impact of symptoms	**P8:** "I feel, honey, more tired than anything else, you know? [...] There's the coughing up blood. It only happens early when I get up."

9 (pg 5)	P9	Expectations during cancer treatment	Impact of symptoms	**P9:** "Some aches and pains, but let's say everyone rates it from 0 to 10, the closest I've ever gotten is, let's say, a 5... And indigestion, which is what's been really bothering me the most."
11 (pg 3)	P11	Expectations during cancer treatment	Impact of symptoms	**P11:** "I'll tell you, ma'am, frankly, thank God, after I had the surgery, I don't feel any symptoms yet. I feel like a very... healthy person!"
12 (pg 3)	P12	Expectations during cancer treatment	Impact of symptoms	**P12:** "Very tired... Pain here, in my chest (shows where) and I'm having trouble swallowing food, drinking water, and eating. [...] And a cough!"
1 (pg 6)	P1	Expectations during cancer treatment	Impact of symptoms	**I.C.D.A.:** "Do you believe these symptoms could interfere with your life? In what way?"<br>**P1:** "Ah, it does interfere, doesn't it? Because we were healthy, and now we find ourselves in this situation."
2 (pg 5)	P2	Expectations during cancer treatment	Impact of symptoms	**P2:** "You can't do it, you have to stay seated."
2 (pg 6)	P2	Expectations during cancer treatment	Impact of symptoms	**P2:** "I don't feel weak, I feel weak (simulates a tired expression), but if I just sit here breathing nicely, there's no problem at all."
3 (pg 8)	P3	Expectations during cancer treatment	Impact of symptoms	**P3:** "Ah, it's a bit of a problem, isn't it, because I don't know what it's going to be like when I go back to my job. As a waiter, you need to listen to the customer a lot; sometimes you repeat the order, but even then, you get something wrong, some detail. And hearing, when you don't listen properly, it takes away your focus a bit, actually, right?"
4 (pg 4)	P4	Expectations during cancer treatment	Impact of symptoms	**I.C.D.A.:** "Could these symptoms interfere with your life?"<br>**P4:** "I don't think so [...] because I take medication so I can eat, which I don't have anymore, I lack the appetite to eat.

5 (pg 3)	P5	Expectations during cancer treatment	Impact of symptoms	**P5:** "At work, you know, you don't know if you'll be able to go back to work, what's going to happen."
6 (pg 4)	P6	Expectations during cancer treatment	Impact of symptoms	**P6:** "I won't be able to take care of things, will I? I'll have to stay in bed more, right?"
		Expectations during cancer treatment	Impact of symptoms	**P9:** "Look, it can sometimes interfere with the emotional system. [...] And look, if I continue the way I am with this muscular support, I believe it will get worse if I don't take action, you understand?"
10 (pg 4)	P10	Expectations during cancer treatment	Impact of symptoms	**P10:** "It's possible, if I get dizzy, because I already have labyrinthitis, and when it attacks, I can't do anything around the house... So, I'm afraid that this might happen, but..."
11 (pg 4)	P11	Expectations during cancer treatment	Impact of symptoms	**P11:** "Look, I also hope it doesn't interfere with my life, you know? Like... well... I don't know. Sometimes it might interfere, I don't know, with the things I always do. If it doesn't interfere, that's much better for me."
12 (pg 4)	P12	Expectations during cancer treatment	Impact of symptoms	**P12:** "It interferes [with the symptoms] ... I think I get tired and can't walk. If I walk too much, if I strain myself, I fall to the ground, I can't take it anymore..."
1 (pg 8)	P1	Perceptions about physical exercise in cancer	Availability to perform physical exercise	**I.C.D.A.:** Do you believe that exercise can be performed during cancer treatment?" **P1:** "I believe so, right?"
2 (pg 10)	P2	Perceptions about physical exercise in cancer	Availability to perform physical exercise	**P2:** "Ah... everything that is good... Everything that is treated in such a healthy way is welcome."
3 (pg 10)	P3	Perceptions about physical exercise in cancer	Availability to perform physical exercise	**P3:** "Everybody will react in a different way. Ah no, sometimes chemotherapy doesn't actually have that much of an effect [...], I think if you can do regular exercise, you'll be stronger when you recover, your rehabilitation will be faster, and I think that's the way to go. [...] It would

				depend more on the reactions you get from the chemo, right?”
5 (pg 3)	P5	Perceptions about physical exercise in cancer	Availability to perform physical exercise	**P5:** "It will depend a lot on the physique."
6 (pg 5)	P6	Perceptions about physical exercise in cancer	Availability to perform physical exercise	**P6:** "Can a person handle doing it? I don't think so. [...] A person gets weak, doesn't they? I already feel terribly weak without doing any exercise, nothing."
6 (pg 7)	P6	Perceptions about physical exercise in cancer	Availability to perform physical exercise	**P6:** "I don't think you can do it."
		Perceptions about physical exercise in cancer	Availability to perform physical exercise	**P7:** "I don't think I can (shakes his head no while answering), it will depend on what the doctor says, if she says I can, I'll do it."
9 (pg 7)	P9	Perceptions about physical exercise in cancer	Availability to perform physical exercise	**P9:** “I’ve always believed that anything you do to relieve the patient’s attention, let’s say, is of utmost importance. Any entertainment that diverts the person’s attention from that pathology or from what is unfavorable to them, you occupy their mind with something else. And for me, I think everything is valid. Now… it won’t be valid if their mind doesn’t allow it, that’s where the great danger lies.”
10 (pg 4)	P10	Perceptions about physical exercise in cancer	Availability to perform physical exercise	**P10:** "I think if you're willing, you can do it, you know... I want to have that willingness."
11 (pg 5)	P11	Perceptions about physical exercise in cancer	Availability to perform physical exercise	**P11:** "I think so, it can be done."
13 (pg 4)	P13	Perceptions about physical exercise in cancer	Availability to perform physical exercise	**P13:** "I don't know what the treatment is, so... I guess it's possible."
2 (pg 10)	P2	Perceptions about physical exercise in cancer	Availability to perform physical exercise	**I.C.D.A.:** "And would you, sir, have the courage to do it? If we talked like that, could we...?"

				**P2:** "Yes, I would... I would fall a few times there, but I would (nodding affirmatively)."
5 (pg 4)	P5	Perceptions about physical exercise in cancer	Availability to perform physical exercise	**P5:** "Right now I don't know if I would do it. [...] Oh, I'm always afraid to do it, because it takes away a person's stability a little, you know [the cancer]."
7 (pg 7)	P7	Perceptions about physical exercise in cancer	Availability to perform physical exercise	**P7:** "Ah, if it's to help, I want it! [...] If it's something mild and I do it, I want to get better. But I (makes a repetitive gesture pointing to herself) think it's not possible. If it's going to be harmful like others say [with chemotherapy]... I don't want to do it."
8 (pg 6)	P8	Perceptions about physical exercise in cancer	Availability to perform physical exercise	**P8:** "Me? If they tell me to do it, then I'll do it, because I want to get better."
9 (pg 11)	P9	Perceptions about physical exercise in cancer	Availability to perform physical exercise	**P9:** From the moment it's a team that's working, I have to meet the team's requirements, right? I can't underestimate that. If I'm not contributing to my own needs, I'm contributing my experience to be applied to other people."
12 (pg 2)	P12	Perceptions about physical exercise in cancer	Availability to perform physical exercise	**P12:** "Well, in that part of therapy [physical therapy], I haven't been through any yet. So, in my case, I can't say... So, what I can say, what seems important to me, is important. It's important! I'm not going to say if I want this one, if I want that other one... Whatever seems important to me, I agree with. So, everything that comes up, in that part, I agree with!"
1 (pg 9)	P1	Perceptions about physical exercise in cancer	Effects of physical exercise	**P1:** "I believe it will give us more encouragement, right? We won't give up."
2 (pg 10)	P2	Perceptions about physical exercise in cancer	Effects of physical exercise	**P2:** "I don't know... (shrugs and shakes head negatively), honestly I don't know... Maybe improve this (points to his head) and the performance [...]

				ah, get up, walk around, don't just sit there."
4 (pg 6)	P4	Perceptions about physical exercise in cancer	Effects of physical exercise	**P4:** "Mainly in the head. [...] Because we don't dwell on bad thoughts, so we think about good things (smiles)."
4 (pg 7)	P4	Perceptions about physical exercise in cancer	Effects of physical exercise	**I.C.D.A.:** "If you, ma'am, exercised, do you think it would help you in this treatment process?"<br>**P4:** "Sim."
6 (pg 6)	P6	Perceptions about physical exercise in cancer	Effects of physical exercise	**P6:** "I think exercise is a bit... feeling pain like that, you know?"
9 (pg 5)	P9	Perceptions about physical exercise in cancer	Effects of physical exercise	**P9:** "I believe it would be like reinforcing the foundation of a house, you know? You have to reinforce the base, I think that's exactly what it entails."
10 (pg 4)	P10	Perceptions about physical exercise in cancer	Effects of physical exercise	**P10:** "It can help, right? Strengthen... the muscles, ah... everything in the body. [...] To give me more resistance, for my body, my treatment, for everything. It can help with a lot of things, right? With the cells, you know, those things, I don't know, the resistance."
11 (pg 5)	P11	Perceptions about physical exercise in cancer	Effects of physical exercise	**P11:** "I think he'll make my body feel better... I don't know if he'll actually alleviate the cancer by moving away from me, you know?"
12 (pg 3)	P12	Perceptions about physical exercise in cancer	Effects of physical exercise	**P12:** "It might help me... maybe ease the tiredness... Ah... the food, maybe it will improve, right? Today, to eat, I have to drink water; I'm drinking water and eating at the same time. Maybe the result of that part will improve. Not on the first day, but it might improve over time..."
14 (pg 6)	P14	Perceptions about physical exercise in cancer	Effects of physical exercise	**P14:** "Ah, so you don't atrophy... I don't think you need to stay still. [...] I don't know, maybe... So, you don't stagnate, you know? Maybe you do some exercise, you give your body a jolt... You rest better afterwards, right? Things like that, you know?"

1 (pg 9)	P1	Perceptions about physical exercise in cancer	Effects of physical exercise	**I.C.D.A.:** "Do you think that would interfere with the symptoms you will experience during the radiation?" **P1:** "Well... let's do it and see, right? To see if there will be any problems, right?"
2 (pg 12)	P2	Perceptions about physical exercise in cancer	Effects of physical exercise	**P2:** "Well, as long as it [the exercise] proves to be good, it will be good."
3 (pg 11)	P3	Perceptions about physical exercise in cancer	Effects of physical exercise	**P3:** "It's because sometimes when you're exercising, and most exercises, you really have to be focused, right?! If you focus more on the exercises and forget about the symptoms, or sometimes the symptoms won't be as strong as if you were focused on just one thing."
3 (pg 12)	P3	Perceptions about physical exercise in cancer	Effects of physical exercise	**P3:** "I think it would actually lessen the symptoms. Now, which symptoms, that's going to be more difficult, right?"
5 (pg 4)	P5	Perceptions about physical exercise in cancer	Effects of physical exercise	**P5:** "Let's make a comparison, like the doctor at the radiotherapy center said. He said that in my case, since I have a cough, I'll probably cough a little more, which is already a symptom... So, if I go for a walk, for example, and I get tired, it will be worse, I think."
6 (pg 6)	P6	Perceptions about physical exercise in cancer	Effects of physical exercise	**P6:** "I think if you get diarrhea, you wouldn't be able to exercise, how are you going to do it? With diarrhea, yes."
6 (pg 6)	P6	Perceptions about physical exercise in cancer	Effects of physical exercise	**P6:** "Ah, I think the person wouldn't be able to handle the treatment, they'd stop halfway through... wouldn't they?"
7 (pg 8)	P7	Perceptions about physical exercise in cancer	Effects of physical exercise	**P7:** "I think it can worsen [the symptoms] if there isn't good follow-up, it can get worse, you know. Now, if it's well monitored and the doctor says I can, I'll do it! [...] I think that, if it's light exercise, it won't interfere, but if it's heavier, it will interfere with fatigue, stomach problems, movement... That's the idea I

				have. [...] I think that instead of helping, it will hinder, right?”
8 (pg 8)	P8	Perceptions about physical exercise in cancer	Effects of physical exercise	**P8:** "I think she'll get better, right? Because when she's exhausted... But then it gets better, right?"
9 (pg 9)	P9	Perceptions about physical exercise in cancer	Effects of physical exercise	**P9:** "Because I think exercise isn't going to solve your digestion problems, right? Your digestion would normally be addressed through diet and nutritional guidance."
11 (pg 6)	P11	Perceptions about physical exercise in cancer	Effects of physical exercise	**P11:** “I think that exercise, along with the treatment, the treatment will help me to get rid of the cancer and the exercise would, how do you say... help me to be more active, to stay in shape, to fight it [the cancer].”
2 (pg 10)	P2	Perceptions about physical exercise in cancer	Modalities of physical exercise in cancer	**I.C.D.A.:** "So for cancer patients, you think it should be something lighter?"<br>**P2:** "I don't know (with a doubtful expression and shaking her head negatively), I can't tell you..."
2 (pg 10)	P2	Perceptions about physical exercise in cancer	Modalities of physical exercise in cancer	**P2:** "I think we'll go all the way to the sidewalk over there and come back."
2 (pg 10)	P2	Perceptions about physical exercise in cancer	Modalities of physical exercise in cancer	**P2:** "Just having a segment, right? How to do it, we'll learn."
2 (pg 13)	P2	Perceptions about physical exercise in cancer	Modalities of physical exercise in cancer	**P2:** "I don't know anything, I don't know how to do it."
3 (pg 12)	P3	Perceptions about physical exercise in cancer	Modalities of physical exercise in cancer	**P3:** "I don't even have the slightest idea [...] But now, what kind of neck exercise would that be (laughs)? Stretching?"
3 (pg 12)	P3	Perceptions about physical exercise in cancer	Modalities of physical exercise in cancer	**P3:** "I think it would be a whole-body treatment, right? Because the dynamics of your body sometimes start in your legs and go practically to your head. So, it's something that will affect you more than if it were just a localized treatment."

7 (pg 7)	P7	Perceptions about physical exercise in cancer	Modalities of physical exercise in cancer	**P7:** "Ah, I don't think it should be anything too heavy, it should be something light, because whoever is undergoing this kind of treatment isn't exactly healthy, right? (laughs) I think it has to be something lighter... because the old lady can't handle anything too heavy! (another laugh)"
7 (pg 7)	P7	Perceptions about physical exercise in cancer	Modalities of physical exercise in cancer	**P7:** "Anything that involves moving my arms or legs... I don't think I can even move my waist because it will hurt my back, right? Besides the herniated disc, there's the surgery... So, I think I'll have to adapt to the type of exercise and I'll have to say how much I can handle... Isn't that, right?"
9 (pg 8)	P9	Perceptions about physical exercise in cancer	Modalities of physical exercise in cancer	**P9:** "I believe that its [exercise] is limited, you know. I believe that you can't be doing everything you want, right? You can't, like, do a triathlon, you know? (laughs) [...] I think the treadmill isn't good, I think it's too much effort. It would be more stationary and also in terms of balance."
9 (pg 9)	P9	Perceptions about physical exercise in cancer	Modalities of physical exercise in cancer	**P9:** "Limiting, of course, I think you have to respect your body's limits. So, your body will say 'no, not this one' or 'not from here on out,' right? You can't get too complacent. I think if you've reached '10,' you have to try to reach '11.' But if you don't reach '11' either, stay at '10,' that's fine."
11 (pg 5)	P11	Perceptions about physical exercise in cancer	Modalities of physical exercise in cancer	**P11:** "It's not one of those exercises that involve lifting weights, you know? I don't think it can be like that for cancer. It must be another type of exercise, right? A dance, something lighter. But then you'll just get used to it, until your body gets used to it."
12 (pg 3)	P12	Perceptions about physical exercise in cancer	Modalities of physical exercise in cancer	**P12:** "I've heard of it, yes. But I don't know what exercise it involves..."

14 (pg 5)	P14	Perceptions about physical exercise in cancer	Modalities of physical exercise in cancer	**P14:** "I don't know, walking on a treadmill, or something like that."
1 (pg 7)	P1	Third-party support	Healthcare professionals involved	**P1:** "The professionals are the doctors, right?"
1 (pg 7)	P1	Third-party support	Healthcare professionals involved	**P1:** "Nurses, and everyone who has been supporting me."
1 (pg 7)	P1	Third-party support	Healthcare professionals involved	**I.C.D.A.:** "Physiotherapist, do you, sir, think this could help? Could you help?"<br>**P1:** "It might help! (looks around thoughtfully) It should help, right?!"
2 (pg 3)	P2	Third-party support	Healthcare professionals involved	**P2:** "Ah, a physiotherapist is interesting, another doctor specializing in I don't know what... well... I honestly don't know..."
2 (pg 8)	P2	Third-party support	Healthcare professionals involved	**P2:** "I know that anyone who could help me would be welcome."
2 (pg 8)	P2	Third-party support	Healthcare professionals involved	**P2:** "Those therapists, doctors, especially, and... I don't know... others I don't know (laughs)."
3 (pg 9)	P3	Third-party support	Healthcare professionals involved	**P3:** “The nutritionist's part will be very helpful, because there will be phases where you won't be able to manage with a normal diet [...] also the psychological part, because your mind has been racing, really! [...] And the dentist, who will be doing laser treatment on your mouth. [...] I don't know (makes a doubtful expression, furrowing her brow) if there are any more.”
4 (pg 2)	P4	Third-party support	Healthcare professionals involved	**P4:** "I think the best doctors (shakes his head in denial). I was very lucky because I went to the dentist and the dentist discovered that I had mouth cancer."
5 (pg 3)	P5	Third-party support	Healthcare professionals involved	**I.C.D.A.:** "Have you ever heard of other fields besides being a doctor or nurse?"<br>**P1:** "No (shrugs)."

6 (pg 3)	P6	Third-party support	Healthcare professionals involved	**P6:** "An oncologist, right? And there are other doctors too, right? I'm already seeing a psychologist too, I've come three times already... she's on vacation now..."
7 (pg 3)	P7	Third-party support	Healthcare professionals involved	**P7:** "Only the ones I'm seeing now, the doctors, and I met the guys from physiotherapy who would come to my room, and so, not that we know of any continuous treatment other than the doctors, right?"
9 (pg 4)	P9	Third-party support	Healthcare professionals involved	**P9:** "Endocrinologist, the oncologist part. [...] So to tell you the truth, it's a lot for so few people, you know? There's nothing more you could want, right? As they say, the only thing more there is God, so that's it. I think we don't need any more professionals, right..."
10 (pg 2)	P10	Third-party support	Healthcare professionals involved	**P10:** "Only God. [...] Because the doctors I used to go to were like... gynecologists, those kinds of doctors... I'm getting to know more doctors now."
10 (pg 4)	P10	Third-party support	Healthcare professionals involved	**I.C.D.A.:** "Do you think everyone here is a doctor?" **P10:** "Yes... everyone who's calling me is a doctor."
1 (pg 4)	P1	Third-party support	Family support	**P1:** "[...] I'm feeling very strong, and my family is supporting me too, right?!"
7 (pg 6)	P7	Third-party support	Family support	**P7:** "My sister goes there with me. They haven't abandoned me for a single day, thank God she stayed with me the whole time at the hospital, during surgery, after surgery, and she's been supporting me. She said, 'I'll only stop when you start chemo and you see that you're doing well, then you can manage as best you can...'"
11 (pg 1)	P11	Third-party support	Family support	**P11:** "I go to several doctors and each one says something different. Since I'm practically illiterate, I can't understand anything, I forget what they say... But my daughter remembers everything!"

2 (pg 7)	P2	Context of the diagnosis	Impact of diagnosis	**I.C.D.A.:** "Before the cancer diagnosis, what were you able to do that you can no longer do now because you're experiencing this cough and shortness of breath?" **P2:** "Nothing else [...] I used to walk, walk calmly, come and go, but today I can't do that anymore."
5 (pg 2)	P5	Context of the diagnosis	Impact of diagnosis	**P5:** "Ah, because we're used to working. Then I've already been away from work, you know. So, I go to my sister's house, and I live on a farm. Sometimes I go to my son's house. So, a lot of things change, if you have to go back and forth, you're not at home."
6 (pg 4)	P6	Context of the diagnosis	Impact of diagnosis	**P6:** "What has changed is that I can't do many things... like, I can't clean a house because it makes me short of breath, if I walk I get short of breath."
6 (pg 5)	P6	Context of the diagnosis	Impact of diagnosis	**P6:** "I wake up feeling good, then I start doing a bunch of things and then it starts... feeling unwell and it lasts all day... until I go to sleep at night."
7 (pg 4)	P7	Context of the diagnosis	Impact of diagnosis	**P7:** "No, I'm not doing anything, am I? [...] I spend most of my time walking around the house, doing nothing! That's my routine, doing nothing! [...] I used to do laundry, iron, cook, teach, I sold costume jewelry, I had a hectic life, my life was crazy. I had three jobs. I handled it all perfectly... I never had a sedentary life, not at all!"
7 (pg 5)	P7	Context of the diagnosis	Impact of diagnosis	**P7:** "I prefer to rest and take care of myself and heal quickly, rather than doing things. I've already tried to clean the kitchen, the side where I cut myself hurts a lot, I've tried to hang clothes on the line and I couldn't. So, I prefer to stay quieter, you know? To do things like clearing the table, putting away dishes... things like that, lighter things."

8 (pg 2)	P8	Context of the diagnosis	Impact of diagnosis	**P8:** “Because before, I would go to the fields, work all day, and feel nothing. And today I can't go. The exhaustion won't let me. I can't even run fifty meters. And it attacked suddenly. If I hadn't gone to the health center, I think I would have already died.”
11 (pg 4)	P11	Context of the diagnosis	Impact of diagnosis	**P11:** “But now I won’t be able to ride [a bicycle]… I won’t be able to ride. When I go out nearby, I ride a little, but now I can’t anymore, because I used to ride a lot… I think I’m afraid of this, of overexerting myself here, you know (points to the tumor on her face)?”
12 (pg 1)	P12	Context of the diagnosis	Impact of diagnosis	**P12:** “Thank God I quit smoking… Because I was hospitalized for almost fifteen days. Then, there came a point there, and I said, as soon as I got there… I got it into my head, you know? I got it into my head and said, ‘Look, I’m not going to go looking for a bottle of liquor, I’m not going to go looking for a cigarette there, because I’m taking medicine, injections, all the time.’ And I told myself, ‘Let go of that.’ And I’ve kept that in mind ever since, thank God!”
2 (pg 8)	P2	Context of the diagnosis	Impact of diagnosis	**P2:** "We miss being around or talking to someone, don't we?"
3 (pg 1)	P3	Context of the diagnosis	Impact of diagnosis	**P3:** "It's something that's affecting us a bit, it's affecting the family, it's affecting our heads a bit, actually, you know?"
3 (pg 6)	P3	Context of the diagnosis	Impact of diagnosis	**P3:** "And the cancer ended up destabilizing things a bit around the family, because the health we used to have, sometimes it was just a cold or something simple that we'd treat at the health center and it would heal. No, today, we go straight into aggressive treatment.”
3 (pg 13)	P3	Context of the diagnosis	Impact of diagnosis	**P3:** "Today it's affecting my family more, you know. My parents, siblings, uncles. Yeah,

				I'm trying to figure out how to talk to my children about it."
14 (pg 6)	P14	Context of the diagnosis	Impact of diagnosis	**P14:** "So, before the [biopsy] results came out, she [my wife] was suffering from very severe depression… She threw alcohol on herself and set herself on fire. She burned to death. Then I got a little discouraged, afterwards… And because of them, her (points to his daughter), her sister, my grandson, I cheered up. I said, "No, I'm going to run, this isn't how it ends." It was really bad. It still is, you know? She was my companion. Instead of her being here (points to his companion), her mother should be… always with me."
3 (pg 3)	P3	Context of the diagnosis	Process for obtaining a diagnosis	**P3:** "Oh, there were a lot of disruptions, there were a lot of things that were like, 'oh, it should have closed a little earlier'."
3 (pg 4)	P3	Context of the diagnosis	Process for obtaining a diagnosis	**P3:** "Sometimes she [my wife] would say that a bad smell was coming from my nose, but it wasn't investigated much, it wasn't given priority, you know? Sometimes it was an imaging exam, sometimes a simple CT scan, that after so much complaining of constant sinusitis would confirm that the cancer was in my sinuses."
3 (pg 6)	P3	Context of the diagnosis	Process for obtaining a diagnosis	**P3:** "He [the doctor] used the term 'we're going to try to cure you' (sad expression). I think that if they're going to try, then there's no certainty about something. Either there's effective treatment in the city, which is a very large city, and it's well-equipped, or they end up transferring us to another region that's more advanced in cancer treatment [...] Because the phrase 'we're going to try' doesn't give you the support of 'you're being taken care of'**.**"
6 (pg 2)	P6	Context of the diagnosis	Process for obtaining a diagnosis	**P6:** "She had a lot of tests done, and they said it was an arrhythmia, and she went to

				the doctor, arrhythmia... she started coughing in March, a cough, cough, cough that wouldn't go away, so I went to a lung specialist... then he did tests and found a lesion in her lung, she only found out through a CT scan, then the doctor said it's [cancer]... she did a biopsy and everything."
7 (pg 2)	P7	Context of the diagnosis	Process for obtaining a diagnosis	**P7:** "The pain I had before the surgery was so horrible that nothing could stop it, I wanted to bang my head against the wall... three months of despair here at home and being treated all wrong, in Rancharia [a city in the interior]... it was awful."
7 (pg 5)	P7	Context of the diagnosis	Process for obtaining a diagnosis	**P7:** "I'd go to the hospital, take pain medication, go home, go to the hospital again, take pain medication, but nobody could figure out what was wrong with me. Then they said I had a kidney problem, and I said (in an authoritative and angry tone), "Since when did my kidney end up under my ribs? My problem is under my ribs... I don't know what it is, you need to find out what it is!" Then, with great difficulty, the doctor took an X-ray of my torso, that's where he saw it, and that's when the rush began."
10 (pg 2)	P10	Context of the diagnosis	Process for obtaining a diagnosis	**P10:** "I discovered this lump (shows where on her neck), this lump has been bothering me since last year. I was only getting ultrasounds, then I got more worried and went to the doctor [surgeon], that's when he said it would be good to remove it to do a biopsy and that's how they found out."
11 (pg 3)	P11	Context of the diagnosis	Process for obtaining a diagnosis	**P11:** "Ah, I saw the doctor, that dentist... Then, I saw the doctor pulling the tooth... Then, when it was getting inflamed, getting inflamed, I looked for the anesthesiologist, and then she pulled the tooth. I also used a lot of antibiotics, antibiotics to

				reduce the inflammation, but it didn't reduce the inflammation, and it kept swelling, swelling more and more, and when I saw it, it was already closing my eye... The tumor was already closing my eye. That's when I went to the doctor to see what it was. Then the doctor did the exam and confirmed: it's cancer."
12 (pg 5)	P12	Context of the diagnosis	Process for obtaining a diagnosis	**P12:** "But the doctor kept saying it was a heart arrhythmia… My heart was enlarged. I went back to him months later and said, 'I'm going to get tested,' and then my heart was normal. I said, 'You [the doctor] didn't say it was an arrhythmia, where is it?' Then he said to have a lung exam, a test… Then he did it and discovered [the cancer]."
3 (pg 4)	P3	Context of the diagnosis	Process for obtaining a diagnosis	**P3:** "Sometimes the population doesn't have all that time, a year and a half, two years, to get that diagnosis. So, the population is lacking, especially the less informed people who don't know where to look for help..."
3 (pg 4)	P3	Context of the diagnosis	Process for obtaining a diagnosis	**P3:** "It would have to be the same in the media (shakes head negatively), uh... sixty days diagnosed? (speaks shaking head negatively), is he in treatment? Because, what if it were more aggressive?"
7 (pg 6)	P7	Context of the diagnosis	Process for obtaining a diagnosis	**P7:** "Ah, it's been over a month already... She [the doctor] came to talk to me, she had already ordered it, so much so that I already had a blood test and nothing came up. I've been waiting like this for over a month..."
10 (pg 3)	P10	Context of the diagnosis	Process for obtaining a diagnosis	**P10:** "I don't know if it's my anxiety, but I'm finding it slow."
